# Taking up Africa’s cancer challenge

**DOI:** 10.2471/BLT.18.020418

**Published:** 2018-04-01

## Abstract

Botswana, Kenya and Rwanda have started to provide cancer care in their national efforts to achieve universal coverage of health services. Tatum Anderson reports.

When two government ministers decided to speak out about their personal battles with cancer in media interviews a few years ago, they helped to spark a major change in the way cancer is viewed and dealt with in Kenya. 

“When they talked about their illness openly, and the needs of cancer patients, the Kenyan public and the political leadership became more aware of the burden of cancer in our country,” says Dr Nicholas Othieno–Abinya, a medical oncologist at the University of Nairobi, who has been involved in bringing about the changes.

“Since then, it has become easier for cancer patients to access treatment,” he says.

It was Peter Anyang’ Nyong’o, then Kenya’s minister for medical services who first described how he discovered he had prostate cancer in 2011 and, a year later, Beth Mugo, then minister of public health and sanitation, explained how she had been diagnosed with breast cancer. According to Kenya’s national cancer register, there were 40 999 new cancer cases and 28 453 cancer deaths in 2012.

Some 8.8 million deaths worldwide due to cancer in 2015 were reported to the World Health Organization (WHO) and, according to data gathered by the International Agency for Research on Cancer’s (IARC), cancer deaths are projected to increase to 13 million in 2030.

The prevention and control of cancer and other noncommunicable diseases (NCDs) is now high on the global political agenda, and will be taken up by a United Nations High-level Meeting expected to take place in September.

Countries are committed to reducing premature deaths due to NCDs by 25% by 2025, under the *Global action plan for the prevention and control of NCDs (2013–2020)* and by one third by 2030, under Sustainable Development Goal 3.4.

However, progress to date has been slow. While people in high-income countries are covered financially for most forms of cancer treatment, people in two out of three countries in sub-Saharan Africa do not have general access to basic cancer services, says Dr André Ilbawi, a cancer control officer at WHO.

“When services are available, childhood acute lymphoblastic leukaemia can usually be treated successfully, reaching 90% survival rates in high-income countries after five years, but less than 20% of children with this cancer in low- and middle-income countries survive,” Ilbawi says.

The burden of cancer increasing in African countries as a result of ageing, chronic infections (human papillomavirus causing cervical cancer and hepatitis C causing liver cancer) and unhealthy lifestyles such as lack of physical activity, obesogenic diets and tobacco use. However, access to cancer services is limited.

Those who can afford it have traditionally sought cancer services abroad. For most populations in African countries cancers have gone undetected, untreated and unpalliated.

That is now beginning to change. Kenya is one of several African countries trying to make cancer services accessible through publicly-funded health insurance schemes, as the country moves towards universal coverage of health services.

“The combination of diagnostic capacity, high-quality surgical treatment, a package of essential medicines and radiotherapy facilities for treating cancer are hardly available in low- and middle-income countries,” Ilbawi says, adding: “When cancer care is available in these countries, too often it is of low quality or unaffordable, resulting in poor clinical outcomes and financial ruin.”

“When cancer care is available in these countries, too often it is of low quality or unaffordable, resulting in poor clinical outcomes and financial ruin.”

André Ilbawi

In 2016, Kenya’s national insurance system took the bold step of providing coverage for radiation therapy and surgery, and four courses of chemotherapy per year at hospitals across the country making these services free at point of care for the 18% of Kenyans covered by the fund.

Kenya has 22 oncologists for a population of 46 million people and increasing their numbers is critical. “We used to send people abroad for oncology training. Now we train them ourselves,” says Othieno–Abinya, who set up a fellowship programme in medical oncology at the University of Nairobi in 2016. A radiation oncology master’s programme and a gynaecology oncology fellowship programme are due to start this year. Other cadres will also be trained, including cancer nurses and radiation physicists, while health-care workers are being trained to detect cancers earlier in the community.

Five new cancer centres are planned beyond the capital Nairobi, where cancer services have been concentrated. Othieno–Abinya and a team of local and external partners are setting one up in Kisumu country, where Nyong’o is now governor, and another in Mombasa.

One of those partners is Dr Ahmed Elzawawy, a clinical oncologist from Suez Canal University in Egypt, and President of the International Campaign for Establishment and Development of Oncology Centers.

Elzawawy advocates health policy change to make cancer services more widely available in countries with limited resources. “Past strategies have not worked, but have been repeated, widening the gap between what is needed and what is provided,” says Elzawawy.

Some countries prioritize cheaper services, such as screening, over expensive ones, such radiotherapy. “After years, oncologists in these countries found that this is not a good priority,” Elzawawy says. “Early detection of cancer without affordable and evidence-based treatment would be frustrating for patients and medical staff and unethical,” 

For Elzawawy, while centres of excellence are important, establishing smaller cancer centres or units attached to local hospitals is the best strategy to reach the most people. Strengthening primary care and role delegation (task shifting) can also help to improve access to cancer diagnosis and treatment, he adds.

Some countries are finding ways to address the shortage of specialists. For example, to increase capacity to detect and diagnose cancer in Nigeria, pathologists are being trained by experts at the University of Birmingham over Skype.

“Evidence-based cancer care can be made more affordable in low- and middle-income countries,” Elzawawy argues, “without compromising the overall outcome to patients. Examples include oral chemotherapy and developing better dosing strategies or altering fractions (a few large doses of radiation are cheaper than many small ones).

As chair of the Global Health Catalyst initiative at the Harvard Cancer Center in the United States of America, Elzawawy and the initiative’s director Dr Wilfred Ngwa from Brigham and Women’s Hospital in Boston, are helping to set up public–private partnerships that have for example, donated radiation machines for the centres in Kisumu and Mombasa 

Some countries are increasing access to palliative care. In 2011, Rwanda became one of the first African countries to pass a law on palliative care. Now certain health professionals, including nurses, may prescribe morphine to patients with advanced cancer. The Rwandan law made it possible to establish a new cadre of home-based care practitioners to deliver palliative care as well as to help patients manage other NCDs at home.

Rwanda is also working on cancer prevention, for example of cervical cancer, which accounts for some 22% of cancers affecting women in WHO’s African region, by rolling out HPV vaccination. However, it takes years for the effect of prevention measures to be felt, and not all cancers can be prevented and need to be detected early.

Cancer treatment is accessible and reimbursed by the Mutuelle de Santé, the national health insurance scheme covering about 90% of Rwanda’s 11.6-million population, as it moves towards universal health coverage. These services are provided at teaching hospitals and Butaro Hospital, a centre of excellence supported by nongovernmental organization, Partners in Health.

“Most cancer patients in Rwanda come forward too late to be treated, so palliation is essential as complementary supportive care,” says Dr Christian Ntizimira, a palliative care specialist. “To make cancer treatment available without the parallel provision of palliative care would be a cruel injustice.”

“Most cancer patients in Rwanda come forward too late to be treated, so palliation is essential,”

Christian Ntizimira

Ntizimira is executive secretary ad interim of the Rwanda Palliative Care and Hospice Organisation that is tasked by the health ministry to establish palliative care services across the country.

Until 2011, opioid pain medication in Rwanda could only be prescribed to people dying in extreme pain or in hospital-based palliative care, due to fears of diversion and addiction.

As director of Kibagabaga district hospital from 2010 to 2013, Ntizimira worked to lift restrictions on prescribing morphine to those in pain, and widened palliative care training to oncologists, nurses at community health posts and community health workers. 

One of the challenges Kenya and Rwanda and other countries in Africa face is that over the last decades the development aid agenda has not focused on noncommunicable diseases.

“Nobody thought about the need to invest in cancer prevention and management. This is slowly changing,” Ilbawi says.

Last year, the World Health Assembly adopted resolution WHA70.12 recommending 22 actions countries can do to integrate cancer prevention and control into their health agendas.

“WHO is advising countries on how to set priorities according to the growing burden of common and treatable cancers to increase the number of people accessing quality cancer care,” Ilbawi says. 

The high and increasing cost of cancer treatment is a global challenge. Cancer medicines were added to the *WHO Model List of Essential Medicines* in 2015 to encourage countries to focus on effective cancer treatments, but not all countries can afford these.

For example, Botswana incorporated 80% of the 2015 WHO list into their national essential medicines list, according to Dr Yehoda Martei from the University of Pennsylvania, USA, who is working with the government.

Trastuzumab, an important medicine in the treatment of breast cancer, would benefit only 3% of Botswana’s cancer patients, but consume 43% of its entire cancer medicines budget. Prioritizing cancer treatments can be particularly challenging in countries with limited health budgets and competing health priorities.

“Many cancer patients face financial ruin when they try to pay for treatment,” Ilbawi says. “We must do more for them. WHO is supporting countries, alongside development partners from different sectors, to make effective cancer prevention and control more widely available.”

**Figure:**
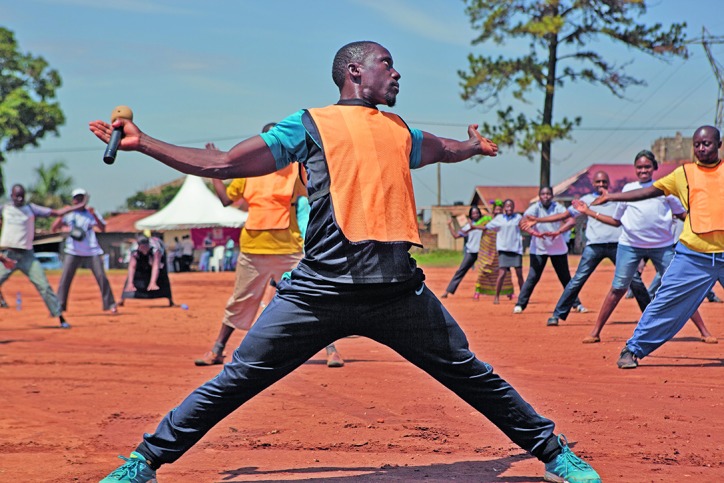
Keeping fit in Uganda helps to prevent noncommunicable diseases including cancer

WHO/A Wang

